# Type II mode of JAK2 inhibition and destabilization are potential therapeutic approaches against the ruxolitinib resistance driven myeloproliferative neoplasms

**DOI:** 10.3389/fonc.2024.1430833

**Published:** 2024-07-18

**Authors:** Sivahari P. Gorantla, Gerin Prince, Jasmin Osius, Dhurvas Chandrasekaran Dinesh, Vijay Boddu, Justus Duyster, Nikolas von Bubnoff

**Affiliations:** ^1^ Department of Hematology and Oncology, Medical Center, University of Schleswig-Holstein, Lübeck, Germany; ^2^ Department of Internal Medicine I, University Medical Center Freiburg, Freiburg, Germany; ^3^ Department of Biochemistry and Molecular Biology, Institute of Organic Chemistry and Biochemistry of the Czech Academy of Sciences, Prague, Czechia

**Keywords:** myeloproliferative neoplasm, JAK2-V617F, ruxolitinib resistance, Hsp90 inhibitior, type I JAK2 inhibition

## Abstract

**Background:**

Ruxolitinib has been approved by the US FDA for the treatment of myeloproliferative neoplasms such as polycythemia vera and primary myelofibrosis. Ruxolitinib will remain a main stay in the treatment of MPN patients due to its effective therapeutic benefits. However, there have been instances of ruxolitinib resistance in MPN patients. As JAK2 is a direct target of ruxolitinib, we generated ruxolitinib-resistant clones to find out the mechanism of resistance.

**Methods:**

Cell-based screening strategy was used to detect the ruxolitinib-resistant mutations in JAK2. The Sanger sequencing method was used to detect the point mutations in JAK2. Mutations were re-introduced using the site-directed mutagenesis method and stably expressed in Ba/F3 cells. Drug sensitivities against the JAK2 inhibitors were measured using an MTS-based assay. JAK2 and STAT5 activation levels and total proteins were measured using immunoblotting. Computational docking studies were performed using the Glide module of Schrodinger Maestro software.

**Results:**

In this study, we have recovered seven residues in the kinase domain of JAK2 that affect ruxolitinib sensitivity. All these mutations confer cross-resistance across the panel of JAK2 kinase inhibitors except JAK2-L983F. JAK2-L983F reduces the sensitivity towards ruxolitinib. However, it is sensitive towards fedratinib indicating that our screen identifies the drug-specific resistance profiles. All the ruxolitinib-resistant JAK2 variants displayed sensitivity towards type II JAK2 inhibitor CHZ-868. In this study, we also found that JAK1-L1010F (homologous JAK2-L983F) is highly resistant towards ruxolitinib suggesting the possibility of JAK1 escape mutations in JAK2-driven MPNs and JAK1 mutated ALL. Finally, our study also shows that HSP90 inhibitors are potent against ruxolitinib-resistant variants through the JAK2 degradation and provides the rationale for clinical evaluation of potent HSP90 inhibitors in genetic resistance driven by JAK2 inhibitors.

**Conclusion:**

Our study identifies JAK1 and JAK2 resistance variants against the type I JAK2 inhibitors ruxolitinib, fedratinib, and lestaurtinib. The sensitivity of these resistant variants towards the type II JAK2 inhibitor CHZ-868 indicates that this mode of type II JAK2 inhibition is a potential therapeutic approach against ruxolitinib refractory leukemia. This also proposes the development of potent and specific type II JAK2 inhibitors using ruxolitinib-resistance variants as a prototype.

## 1 Introduction

JAK2 is an important cytoplasmic tyrosine kinase that plays a major role in the normal development of hematopoiesis and cytokine-mediated signaling (1, 2). The occurrence of somatic activation mutation (valine to phenylalanine) in the pseudokinase domain (V617F) of JAK2 has been implicated in myeloproliferative neoplasms (MPNs) like polycythemia vera (PV: 90% of patients), essential thrombocythemia (ET: 50% of patients) and primary myelofibrosis (PMF: 50% of patients) (3–6). In addition to MPNs, JAK2-V617F mutation appeared at very low frequencies in myelodysplastic syndrome, chronic myelomonocytic leukemia (3-8%), and very rarely in systemic mastocytosis (7, 8). Subsets of PV patients negative to V617F mutation showed a gain of function mutations affecting the exon 12 of JAK2 (9). Other novel mutations located in the JH2 domain are also reported in several hematological malignancies including D620E in PV patients (10), C661Y in unclassified MPN (11), L611S in ALL (12), and IREED in Down syndrome (13). Biochemical studies have shown that all these mutations lead to constitutive activation of JAK2. In addition to the point mutations, JAK2 is also involved as a fusion protein due to chromosomal translocation. A t (9, 12) (p24: p13) leads to the generation of *TEL::JAK2* fusion associated with the development of T-cell childhood acute lymphoblastic leukemia (14, 15). Wild-type JAK2 signaling is also involved in some solid tumors such as breast cancer (16). Taken together, these discoveries encouraged the development of small molecular inhibitors against JAK2. Several JAK family kinase inhibitors have been developed and are currently tested in preclinical and clinical studies (17). Among those, ruxolitinib and fedratinib have been approved for the treatment of intermediate and high-risk myelofibrosis, while ruxolitinib was also approved for PV patients intolerant to hydroxyurea. Unlike imatinib in chronic myeloid leukemia (CML), where already 6 months of TKI treatment can result in a durable clinical response by reduction of the *BCR::ABL1* transcript, JAK2 inhibitor short-term treatment does not induce a significant reduction in MPN-driving allele burden (18, 19). Nevertheless, long-term studies on ruxolitinib indicated a reduction of the mutant allele burden, improvement of bone marrow fibrosis, and increase in overall survival (20–23). Due to these benefits, ruxolitinib remains a mainstay for the treatment of MPN patients. However, it becomes evident from clinical trials that JAK2 inhibitor treatment has a limited effect on disease-driving stem cells and thus, it is unlikely that these inhibitors induce complete remission in MPN patients (24). In addition to ruxolitinib and fedratinib, lestaurtinib is a JAK2-specific inhibitor that inhibits expanded erythroid cells in PV patients. Compared to ruxolitinib, lestaurtinib showed modest clinical recovery with improvement of spleen size and no improvement in bone marrow myelofibrosis and JAK2-V617F allele burden (25). In CML, NSCLC and GIST, it has been demonstrated that acquired resistance to imatinib is due to the emergence of secondary resistance mutations in the target kinase (26–28). In the case of *BCR::ABL*, Azam et al. demonstrated that more than 60 residues in the kinase domain are involved in the resistance against ABL kinase inhibitor imatinib (29). These results led to the development of second and third generation kinase inhibitors in the CML in order to treat the disease efficiently. So far, no inhibitor resistant JAK2 mutations have been reported in patients, although ruxolitinib has been used for more than ten years in the clinic.

Current JAK2 inhibitors such as ruxolitinib, fedratinib and lestaurtinib are type I kinase inhibitors that bind to the active conformation (“DFG-in” state) of JAK2 when the activation loop tyrosines (Tyr1007/Tyr1008) are phosphorylated. In contrast, type II inhibitors bind JAK2 in the inactive (“DFG-out” state) conformation. Previously, Meyer et al. study demonstrated that type II JAK2 inhibitor CHZ868 is highly potent against the type I inhibitor persistent clones, which do not have JAK2 mutations (30). However, the potency of type II JAK2 inhibition against the type I resistant JAK2 variants has not been shown in the JAK2-V617F model. In order to predict the drug resistant mutations against the type I JAK2 inhibitor ruxolitinib and to evaluate the type II JAK2 inhibition role in these variants, we used a cell-based screening strategy. In this study, we have used ENU- (ethyl-nitrosourea) a chemical mutagenesis method to detect the critical residues which mediates the strong resistance against the ruxolitinib. Identification of these residues will be important for the development of next-generation JAK-family kinase inhibitors with better therapeutic efficiency as JAK-family kinases play a major role in several hematological malignancies. Using this method, we were able to identify seven different mutations in the kinase domain of JAK2 that induce strong ruxolitinib resistance. We also evaluated the effect of other JAK2 inhibitors such as fedratinib, lestaurtinib, and CHZ-868 towards the ruxolitinib-resistant variants. Finally, our study also provides evidence that HSP90 inhibitors are potent against ruxolitinib-resistant variants.

## 2 Materials and methods

### 2.1 Cell culture and DNA constructs

Ba/F3 cells (CVCL-0161) were obtained from the German Resource Centre for Biological Material (DSMZ). Ba/F3 cells were maintained in RPMI 1640 (Gibco, Billings, MT, USA) medium containing 10% fetal calf serum in the presence of 2ng/ml murine IL-3. These cells were transfected by retroviral gene transfer and transformed upon withdrawal of IL-3. Phoenix E helper-virus-free ecotropic packaging cells (CVCL-H717) (a kind gift from Dr. G. Nolan, Stanford, USA). All cell lines were tested and confirmed mycoplasma-free. JAK2 mutations were introduced in MSCV-EYFP-V617FJAK2 using the QuikChange mutagenesis kit (Stratagene, Amsterdam, The Netherlands). Total RNA was extracted with TRIzol reagent (Invitrogen, Carlsbad, CA, USA). For RT-PCR of JAK2 encompassing the kinase domain, the following primers are used. JAK2 RT–KD for 5’-gaaaatgacatgttaccaaatatg-3’ and JAK2 RT-KD rev 5’-ggagtaaacaaactgttaaag-3’. For sequencing the kinase domain, the following primers were used: 5’-ctagggttttctggtgcctttgaag-3’ and 5’-gggcgttgatttacattattgttcc-3’.

### 2.2 Cell line authentication

All cell lines mentioned above were recently authenticated using short tandem repeat (STR) analysis. Validation was performed by Microsynth GmbH, Göttingen, Germany. Profiles are available upon request.

### 2.3 Inhibitors

Ruxolitinib was a kind gift from Novartis Pharma AG, Basel, Switzerland. Fedratinib was purchased from Selleckchem (Houston, USA). Lestaurtinib and CHZ-868 were purchased from Calbiochem. 17-AAG and Geldanamycin were purchased from Sigma-Aldrich (Taufkirchen, Germany). All the inhibitors were dissolved in dimethyl sulfoxide to make stock solutions of 10mM and stored at –20°C.

### 2.4 Generation of drug-resistant variants

The selection of ruxolitinib-resistant clones was described previously (31). Briefly, Ba/F3 cells expressing JAK2-V617F cells were pretreated with ENU and cultured in 96–well plates at a density of 4 x 10^5^ cells per well in the presence of ruxolitinib at indicated concentrations. Colonies that became visible after 14 to 20 days in the respective wells were picked, expanded, and analyzed. Ba/F3 MSCV-EYFP-JAK2-V617F cells were pretreated with chemical mutagen N-ethyl-N-nitrosourea (ethyl nitrosourea) twice for 12h at a concentration of 50μg/ml. Resulting inhibitor-resistant sublines were cultured in the presence of ruxolitinib at a concentration corresponding to that used during the screen. After ENU treatment, cell viability and phenotype were analyzed with FACS analysis.

### 2.5 Proliferation assay

Proliferation was measured using an MTS (3-(4,5 dimethylthiazol-2-yl)-5-(3-carboxymethoxyphenyl)-2-(4-sulfophenyl)-2H-tetrazolium)-based method by absorption of formazan at 490nm (CellTiter 96; Promega, Madison, WI). Measures were taken as triplicates after 48 and 72 hours of culture without cytokines, as described previously (32). The IC50 value was calculated using Prism Software.

### 2.6 Western blot

Ba/F3 cells were cultured for 2.5 hours without and in the presence of inhibitor at the indicated concentrations. Cell lysis, sodium dodecyl sulfate-polyacrylamide gel electrophoresis (SDS-PAGE), and immunoblotting were done as described previously (33). JAK2, pSTAT5, and phosphotyrosine antibodies were purchased from Upstate Biotechnology (4G10 and PY20) (Biozol, Eching, Germany). STAT5 and pJAK2 antibodies were obtained from Santa Cruz Biotechnology (Heidelberg, Germany). Bands were visualized using the enhanced chemiluminescence (ECL) system (Amersham, Braunschweig, Germany).

### 2.7 Protein and ligand preparation

The high-resolution 3D structure of human tyrosine-protein JAK2 kinase JH1 domain (PDB ID: 7LL4) with exceptional resolution 1.31Å, was directly retrieved from the protein data bank (PDB), selected specifically for the *in-silico* analysis. Alongside the wild-type structure of the JAK2 JH1 domain, three mutants (L902Q, Y931C, and L983F) were engineered using Maestro within the Schrodinger suite following standard protocols. All variants of the JAK2 JH1 domain structure, both wild-type and mutant, underwent optimization and successive minimization steps until reaching a 0.30Å convergence, employing the OPLS-3e force field. Simultaneously, the ligands ruxolitinib, fedratinib, lestaurtinib, and CHZ-868 were retrieved from PubChem. These ligands underwent conformational generation under standard pH conditions, generating up to 32 conformations per ligand, followed by minimization using the OPLS-3e force field.

### 2.8 Molecular docking simulation

The prepared wild-type and mutant proteins underwent active site prediction using the sitemap module, and the predicted sites were crosslinked with co-crystal-bound ligand structure. Subsequently, the predicted sites were manually picked for the Glide-based grid generation to position the ligands to be docked, including co-crystal ligands. The grid box is set at 2Å from the center radii. Successful grids for wild-type and mutant proteins enable docking with prepared ligands utilizing the eXtra Precision mode (XP) docking in the Glide module. The final binding positions were validated through MM/GBSA calculations. The best scoring pose, indicating optimal bonding, was visualized using the Maestro visualizer, and the corresponding scores were documented.

### 2.9 Statistical analysis

Values are represented as mean SEM. The comparison of multiple groups was analyzed by one-way ANOVA test and the comparison between two groups was analyzed by unpaired *t* test. **p<0.01, *p<0.05 and n.s., not significant, p>0.05 by Student’s *t* test. **p<0.005, ****p<0.0001 were considered for one one-way ANOVA test.

## 3 Results

### 3.1 Frequency of drug-resistant clones is decreased after increasing the ruxolitinib concentration

**Figure 1:**
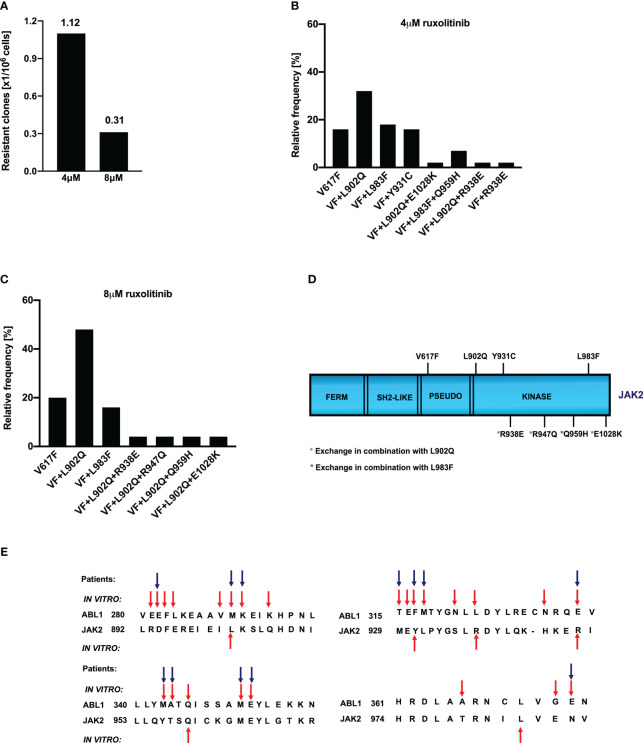
Spectrum and relative frequency of the mutations identified in the ruxolitinib screen: Single clones of Ba/F3 cells growing in 96-well plates in the presence of 4μM and 8μM ruxolitinib were picked and analyzed for the presence of JAK2 kinase domain mutations. Shown is the frequency of resistant clones per million cells **(A)**. Resistant clones grown in the presence of 4μM ruxolitinib were expanded and analyzed for kinase domain mutations. Shown are the relative frequency of each mutation in 4μM ruxolitinib concentration **(B)**. Similarly, relative frequency of mutations in 8μM ruxolitinib concentration is shown **(C)**. Location of the putative JAK2 inhibitor resistant mutations is shown **(D)**. Alignment of homologous regions in JAK2 and ABL1 **(E)**. The blue arrows indicate the mutations identified in *BCR*::*ABL* reported to confer imatinib resistance in patients. Red arrows indicate the mutations identified by the *in vitro* mutagenesis screening method.

Ruxolitinib is a potent JAK2/JAK1 specific inhibitor that exhibits remarkable clinical activity against the JAK2-V617F mediated MPNs (23). In the case of several hematological malignancies, it has been demonstrated that resistance is due to the acquisition of point mutations in the target kinase. However, in the case of JAK2-mediated MPNs, none of the patients displayed mutations in JAK2 kinase, even though persistent to ruxolitinib therapy. The mechanism responsible for ruxolitinib persistence in MPN patients has yet to be demonstrated. To understand the ruxolitinib persistence, we developed a cell-based screening strategy against the 1μM, 2μM ruxolitinib. Surprisingly, none of the resistant clones growing at these concentrations display mutations in the JAK2 kinase domain as shown previously in our lab (34). Since resistant clones did not display point mutations in JAK2, we decided to perform resistance screening with an ENU (ethyl-nitrosourea: a chemical mutagen) pretreatment before the ruxolitinib exposure. At 4μM ruxolitinib concentration, which approximates the maximum measured plasma concentration, the frequency of resistant clones was 1.12 with a million cells input (**Figure 1A**). The frequency of resistant clones decreased in 8µM ruxolitinib concentration and was limited to 0.3 per million cells input (**Figure 1A**).

### 3.2 L902Q, Y931C, and L983F are the most frequent mutations identified in the ruxolitinib screen performed at 4 μM and 8 μM concentrations

To determine whether the drug resistance is due to the acquisition of point mutations in the kinase domain of JAK2, we sequenced the drug-resistant clones from 4μM and 8μM concentrations. Analysis of 4μM drug-resistant clones revealed L902Q, L983F, and Y931C are the most frequent mutations with a relative frequency of 33%, 16%, and 16%, respectively (**Figure 1B**) whereas resistant clones growing in the presence of 8μM ruxolitinib displayed L902Q and L983F with high abundance (44% and 18%, respectively) (**Figure 1C**). In addition to these two mutations, some of the resistant clones displayed compound mutations such as L902Q+R938E, L902Q+R947Q, L902Q+E1028K, and one resistant clone displayed compound mutation together with L983F (L983F+Q959H) in 8μM ruxolitinib (**Figure 1C**). Similar to the 8μM resistant clones, 4μM resistant clones also displayed compound mutations. Among those, L902Q+R938E and L983F+Q959H were identified in the 4μM resistant screen (**Figures 1B, D**). One clone displayed the R938E mutation alone (**Figures 1B, D**). Analysis of the sequencing results suggests that all the compound mutations are in cis-fashion in drug-resistant clones. Approximately, 20 percent of resistant clones did not display any mutations in the kinase domain of the JAK2 except the V617F mutation (**Figures 1B, C**). These results suggested that ruxolitinib exposure to the JAK2-V617F cells leads to the generation of JAK2 variants in the cell-based method. To determine whether these mutations are valid in the clinical settings, we checked the homologous mutations in *BCR::ABL* (**Figure 1E**). The most abundant mutation, L902Q, in JAK2 is homologous to M290 (C-helix) of *BCR::ABL*, which is involved in imatinib resistance (29, 35). JAK2-Y931 is homologous to F317 in ABL1 and has also been associated with imatinib resistance in CML patients (29, 36). JAK2-Y931 is located in the adenine-binding region of the hinge and is involved in direct interaction with the inhibitor. Consistent with our results, previous data also suggest that JAK2-Y931C has been identified in a resistant screen generated against the JAK2-inhibitor BVB808 (37). These results indicate that Y931 residue is ATP-competitive inhibitor specific. Finally, L983 of JAK2 is homologous to L370 of *BCR::ABL*. The leucine at this position is highly conserved among the tyrosine kinases, but so far, none of the kinases have shown drug resistance by changing this residue. In addition to this, we also found that JAK2-Q959 is homologous to the Q346 of *BCR::ABL* which is reported as an imatinib-resistant variant (29) (**Figure 1E**).

### 3.3 Drug-resistant mutations identified in the cell-based screen transformed Ba/F3 cells and showed constitutive JAK2-STAT5 activation

**Figure 2:**
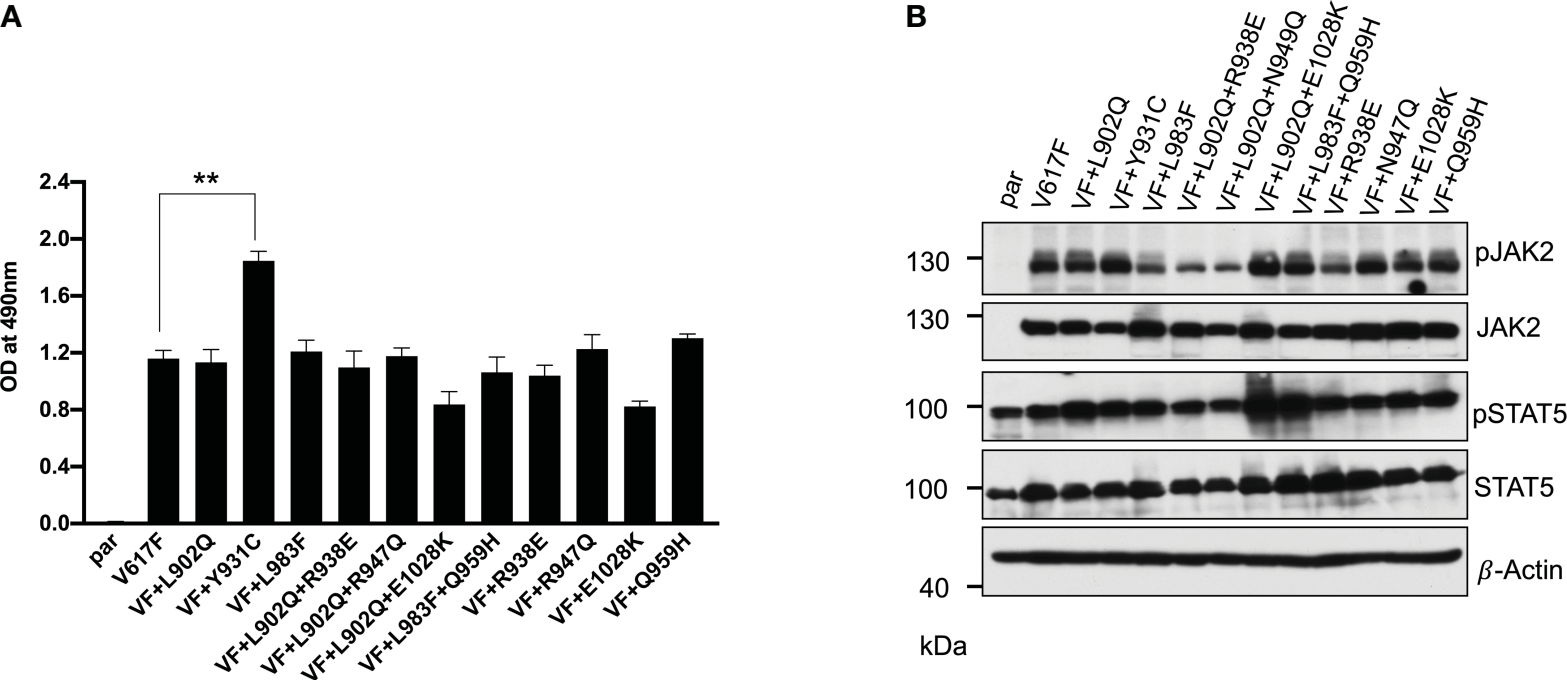
Resistant mutations identified in the ruxolitinib screen transform the Ba/F3 cells and display constitutive activation of JAK2 and STAT5: Mutations identified from 4 and 8μM screen were cloned in JAK2-V617F background either as single mutation or compound mutations and stably expressed in Ba/F3 cells by retroviral gene transfer method. The transformation ability of the mutant JAK2s was measured using MTS- based method after 72hrs without IL-3 (n=3). JAK2-Y931C showed enhanced cell growth compared to JAK2-V617F, whereas the remaining JAK2 mutants showed equal transformation ability **(A)**. **p<0.01. Western blot analysis was performed using Ba/F3 cells expressing the JAK2 mutants and measured the activation of JAK2 and STAT5 **(B)**. A representative image of n=2 two independent experiments is shown.

In the case of *BCR::ABL*, mutations in the kinase domain of the ABL not only confer the resistance but also increase the kinase activity (38). To check this possibility in the case of JAK2, we cloned all the mutants in the V617F background and stably expressed the mutants in the Ba/F3 cell line. Concurrent expression of JAK2-V617F in the Ba/F3 cell line confers the IL-3 independent cell growth, as reported previously (3, 32). JAK2-V617F+L902Q, JAK2-V617F+L983F, JAK2-V617F+L902Q+R938E, JAK2-V617F+L902Q+R947Q, JAK2-V617F+L902Q+E1028K and JAK2-V617F+L983F+Q959H were able to give IL-3 independence to the Ba/F3 cell line (**Figure 2A**). Compared to JAK2-V617F, JAK2-V617F+Y931C gives enhanced cell growth to the Ba/F3 cell line (**Figure 2A**). Similarly, as expected JAK2-V617F+R938E, JAK2-V617F+R947Q, JAK2-V617F+Q959H, and JAK2-V617F+E1028K gave IL-3 independence to the Ba/F3 cell line. We analyzed the activation of the JAK2-STAT5 axis in JAK2 mutants expressing Ba/F3 cell lines and found that all the mutants could activate JAK2 and STAT5 due to the presence of V617F mutation (**Figure 2B**). Later, we hypothesized that kinase domain mutations alone could activate the JAK2 and give the cytokine-independent growth to the Ba/F3 cells without V617F mutation. To check this, we cloned the L902Q and L983F mutations alone in JAK2 and compared the cytokine-independent growth with JAK2-V617F+L902Q and JAK2-V617F+L983F. However, JAK2-L902Q and JAK2-L983F alone fail to transform the Ba/F3 cells (**Supplementary Figures S1A, B**). These results indicate that V617F mutation is indispensable for IL-3 independent growth and kinase domain mutations alone might not be able to promote cytokine-independent growth to Ba/F3 cells.

### 3.4 Kinase domain mutations confer the ruxolitinib resistance and showed persistent activation of STAT5 at higher concentrations of drug

**Figure 3:**
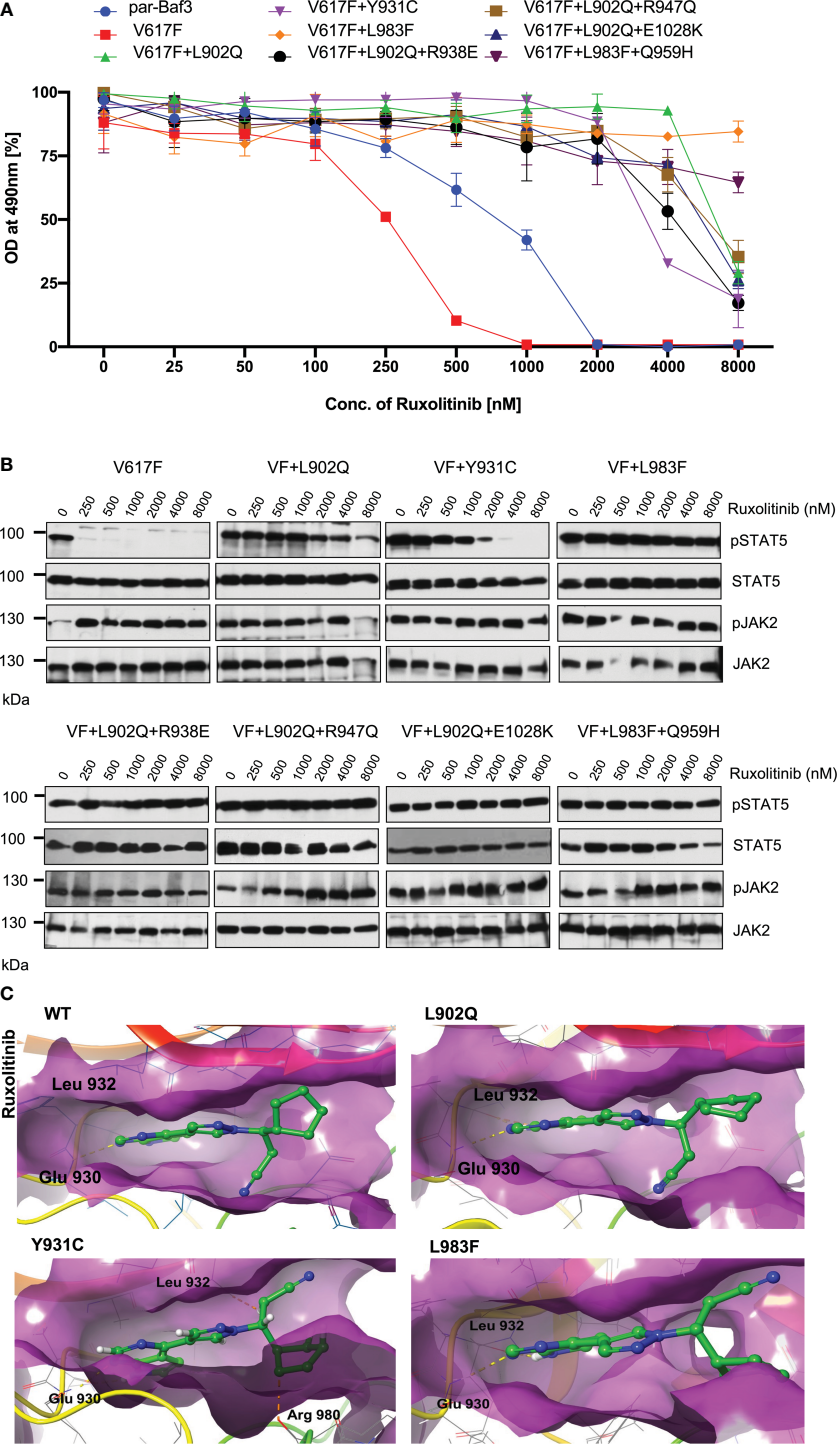
JAK2 kinase domain mutations L902Q, Y931C and L983F displayed resistance phenotype towards the ruxolitinib: JAK2 mutants that were identified with the ruxolitinib including single constituents of compound mutations were recreated in JAK2-V617F using site-directed mutagenesis. Constructs were stably expressed in Ba/F3 cells. Proliferation was measured using 3-(4,5-dimethylthiazol-2-yl)-5-(3-carboxymethoxyphenyl)-2-(4-sulfophenyl)-2h-tetrazolium (MTS)- based method after incubation for 48hrs without and in the presence of increasing concentration of the inhibitor ruxolitinib **(A)**. Data is shown as mean ± standard deviation (SD) (n=3). OD – optical density. Immunoblot analysis of Ba/F3 cells expressing JAK2 mutants cultured with the indicated concentration of ruxolitinib (0, 250, 500, 1000, 2000, 4000 and 8000nM) for 4hrs and lysates were subjected to indicated antibodies **(B)**. A representative image of n=2 two independent experiments is shown. Molecular docking studies were performed using the Glide package of Schrodinger maestro software. The binding interactions of ruxolitinib (stick representation in green carbon) with the JAK2 Kinase domain and its mutants (secondary structure representation as a cartoon) are displayed. Hydrogen bonds are represented by dotted yellow lines, carbon centered hydrogen bonds are represented by dotted orange lines and the amino acids responsible for these interactions are labelled in black. The binding interactions of ruxolitinib with wild type JAK2, L902Q, Y931C and L983F **(C)**.

To determine that these mutations can confer drug resistance, first, we checked the JAK2 mutant cell growth in the presence of increasing concentration of ruxolitinib. JAK2-V617F Ba/F3 cells were sensitive to ruxolitinib (IC50 ~182nM). All the mutants identified in the cell-based screen grew in the presence of high ruxolitinib concentration. V617F+L902Q, V617F+L902Q+R938E, V617F+L902Q+R947Q, V617F+Y931C and V617F+L902Q+E1028K are resistant to ruxolitinib (IC50 > 4000nM) (**Supplementary Table S1**) (**Figure 3A**). V617F+L983F and V617F+L983F+Q959H showed higher resistance (IC50 > 8000nM) (**Supplementary Table S1**) (**Figure 3A**). Next, we sought to determine whether drug-resistant mutants could show the persistent activation of JAK2 and STAT5 in the presence of ruxolitinib. Consistent with the cell proliferation data, western blot data also showed persistent STAT5 activation in all the mutants in higher concentrations of ruxolitinib except JAK2-V617F (**Figure 3B**). At 250nM ruxolitinib concentration, JAK2-V617F displayed the complete absence of STAT5 activation (**Figure 3B**) whereas JAK2-V617F+Y931C showed inhibition of STAT5 at 2000nM concentration, however remaining JAK2 variants did not show decrease STAT5 activation even at 8000nM (**Figure 3B**). As shown before, JAK2 is hyperphosphorylated in the presence of ruxolitinib compared to untreated cells. These results are consistent with the previous report that activation loop phosphorylation (Tyr1007/1008) by JAK inhibitor is mode dependent and treatment with ATP-competitive inhibitors leads to JAK2 hyperphosphorylation (39) (**Figure 3B**). These results indicate that ruxolitinib-resistant variants identified in the cell-based screen method are indeed resistant to higher concentrations of ruxolitinib and keep persistent activation of STAT5.

The binding of ruxolitinib to the JH1 domain of the JAK2 kinase was modeled using the Glide package of Schrodinger maestro software to understand the structural consequences of mutations conferring resistance to ruxolitinib. Regarding the WT, ruxolitinib fits well into the ATP-binding pocket of JAK2, as 91% of its solvent-accessible surface area is buried in the complex. The pyrrolopyrimidine moiety and the pyrazol ring interact favorably with the deep ATP-binding groove (**Figure 3C** wild type). In the present ruxolitinib-JAK2 model, the backbone carbonyl group of Glu 930 accepts a hydrogen bond from the pyrrole ring, whereas the amide group of Leu 932 in the hinge region forms a hydrogen bond with the pyrimidine ring (**Figure 3C**). Additional polar contacts are found i) between backbone atoms of Lys 857 and Gly 858 and the cyclopentyl and nitrile groups, ii) between the side chains of Arg 980 and Asn 981 and the cyclopentyl ring, and iii) between the side chain of Asp 994 and the propanenitrile moiety. The drug is held by numerous hydrophobic interactions with residues Leu 855, Val 863, Ala 880, Val 911, Met 929, Leu 932, and Leu 983 that line the binding pocket (**Figure 3C**). The aromatic ring of Tyr 931 is close enough to the pyrimidine ring to have π-π interaction. Surprisingly, this important interaction does not seem to be critical for the orientation of the inhibitor in the binding pocket because the Y931C mutation does not lead to a different binding pose. There are only minor differences in the ruxolitinib-JAK2 interactions for WT and the Y931C mutation (**Figure 3C** wild type and Y931C). Ruxolitinib forms hydrogen bond interactions with Glu 930 and Leu 932 with wildtype JAK2 domain and these interactions were consistent in all the variants except for the Y931C variant where an additional carbon-centered hydrogen bond was observed with Arg 980 (**Figure 3C**; Y931C). Leu 902 does not directly interact with ruxolitinib. However, it is close to the binding pocket and its mutation to Gln with a polar side chain significantly disturbs the binding of the inhibitor (**Figure 3C**; L902Q). While it remains almost completely buried, the propanenitrile and cyclopentyl moieties essentially exchange their positions, thus leading to unfavorable interactions between the Asp 994 side chain and the cyclopentyl ring. Mutation of Leu 983 to Phe disrupts important hydrophobic interactions (e.g., Ala 880, Val 911, Met 929) with the pyrrolopyrimidine moiety and induces aromatic-aromatic interaction between the new phenyl ring and the pyrrol and pyrazol rings (**Figure 3C**; L983F). Due to altering the shape of the protein’s binding pocket, mutant L902Q, Y931C, and L983F alleles can hinder the drug’s physical fit by disrupting crucial chemical interactions between the drug and the protein, weakening their association and making drug detachment easier (**Supplementary Table S2**).

### 3.5 Ruxolitinib-resistant JAK2-L983F is sensitive towards fedratinib

**Figure 4:**
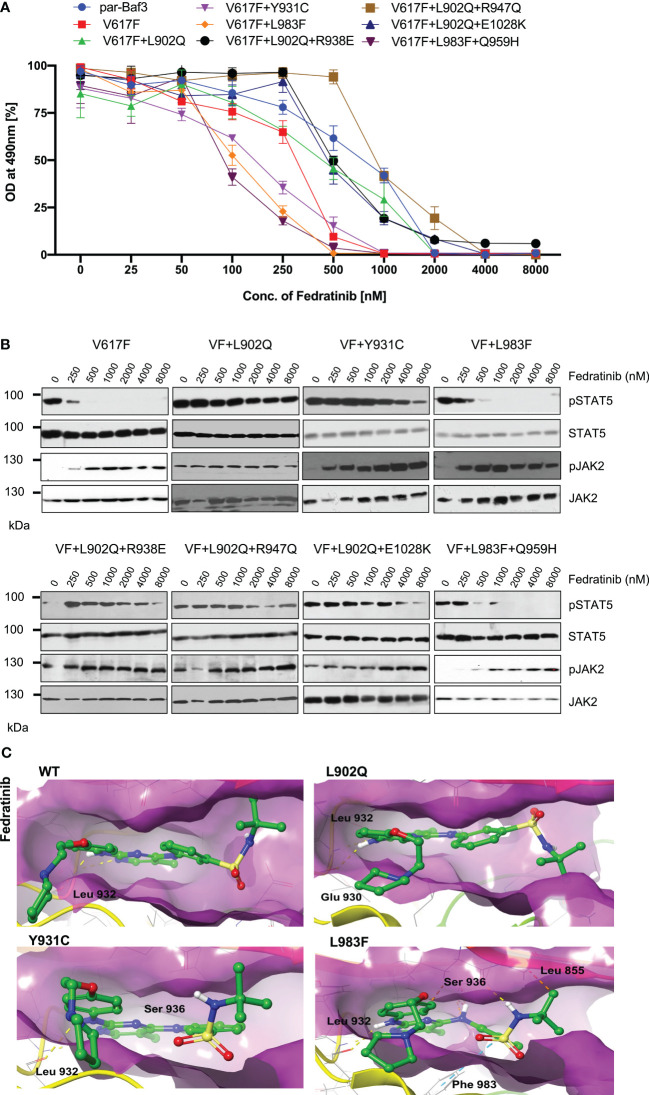
L983F and Y931C display sensitivity towards fedratinib: Mutations that emerged in the ruxolitinib screen were reengineered and expressed in the Ba/F3 cells. Cells were incubated in the presence of fedratinib. Proliferation was measured using 3-(4,5-dimethylthiazol-2-yl)-5-(3-carboxymethoxyphenyl)-2-(4-sulfophenyl)-2h-tetrazolium (MTS)- based method after incubation for 48hrs in the presence of increasing concentration of the inhibitor fedratinib **(A)**. Data is shown as mean ± standard deviation (SD) (n=3). OD – optical density. Immunoblot analysis of Ba/F3 cells expressing JAK2 mutants cultured with increasing concentrations of fedratinib (0, 250, 500, 1000, 2000, 4000 and 8000nM) for 4hrs and lysates were subjected to indicated antibodies **(B)**. A representative image of n=2 two independent experiments is shown. Molecular docking studies were performed using the Glide package of Schrodinger maestro software. The binding interactions of fedratinib (stick representation in green carbon) with the JAK2 Kinase domain and its mutants (secondary structure representation as a cartoon) are displayed. Hydrogen bonds are represented by dotted yellow lines, carbon centered hydrogen bonds are represented by dotted orange lines, pi-pi interactions are represented by dotted cyan lines and the amino acids responsible for these interactions are labelled in black. The binding interactions of fedratinib with wild type JAK2, L902Q, Y931C and L983F **(C)**.

Next, we analyzed for the cross-drug resistance by using another ATP-competitive inhibitor of JAK2, Fedratinib. Similar to the ruxolitinib, JAK2-V617F is sensitive towards fedratinib (IC50 ~172nM). V617F+L902Q, V617F+L902Q+R938E, V617F+L902Q+R947Q, and V617F+L902Q+E1028K are resistant to fedratinib by increasing their cellular IC50 value to more than 1000nM. In contrast, V617F+Y931C is sensitive towards fedratinib (IC50~ 145nM). V617F+L983F and V617F+L983F+Q959H are inhibited at lower fedratinib concentrations (IC50~88nM and 91, respectively) (**Figure 4A**, **Supplementary Table S1**). Consistent with the cell proliferation data, western blot analysis suggested JAK2-V617F showed inhibition of STAT5 activation in the presence of 250nM concentration of fedratinib (**Figure 4B**). Whereas JAK2-V617F+L902Q, JAK2-V617F+Y931C, JAK2-V617F+L902Q+R938E, JAK2-V617F+L902Q+R947Q, and JAK2-V617F+L902Q+E1028K mutants showed persistent activation of STAT5 even at 2000nM concentration of fedratinib (**Figure 4B**) suggesting that these variants are resistant towards fedratinib (**Supplementary Table S1**). Similar to the cell proliferation data, western blot data also suggested that JAK2-V617F+L983F and JAK2- V617F+L983F+Q959H showed inhibition of STAT5 at 500nM concentration of fedratinib (**Figure 4B**). This observation suggests that fedratinib has a better inhibitory profile compared to ruxolitinib as previously suggested (40). Inhibition of STAT5 activation in JAK2-V617F+Y931C was observed at 500nM concentration, suggesting that this variant might be moderately resistant towards fedratinib. Consistent with the previous data, JAK2 phosphorylation (Tyr 1007/1008) was strikingly increased in the fedratinib sensitive mutants (V617F, V617F+Y931C, V617F+L983F and V617F+L983F+Q959H) compared to fedratinib insensitive mutants such as V617F+L902Q, V617F+L902Q+R938E, V617F+L902Q+R947Q and V617F+L902Q+E1028K (**Figure 4B**).

To gain a further understanding of fedratinib sensitivity towards the L983F variant, we performed molecular docking analysis. Fedratinib interacts with wild-type JAK2, L902Q, Y931C, and L983F variants by forming two hydrogen bonds with amino acid Leu 932 (**Figure 4C**). In addition, fedratinib showed - interactions with Phe 983 and hydrogen bond interactions with Ser 936 and Leu 855 in the case of the L983F variant, suggesting stronger binding affinity consistent with the low IC50 (**Figure 4C**; L983F). In contrast to L983F, Y931C could not form additional interaction with Leu 855, which explains the moderate resistance phenotype of Y931C against fedratinib (**Figure 4C**; Y931C). Binding energy analysis displayed both L902Q and L983F showed similar glide scores. However, L902Q is completely resistant to fedratinib (**Supplementary Table S2**). These results are in line with previous findings by Kesarvani et al., where fedratinib binds substrate binding pocket with higher affinity than the ATP site in contrast to the ruxolitinib, which preferentially binds to the ATP site (40). These results suggest that fedratinib might be effective in the suppression of ATP site mutations generated by ruxolitinib due to its ability to bind additional substrate binding sites.

### 3.6 Lestaurtinib is not potent against the ruxolitinib-resistant variants

**Figure 5:**
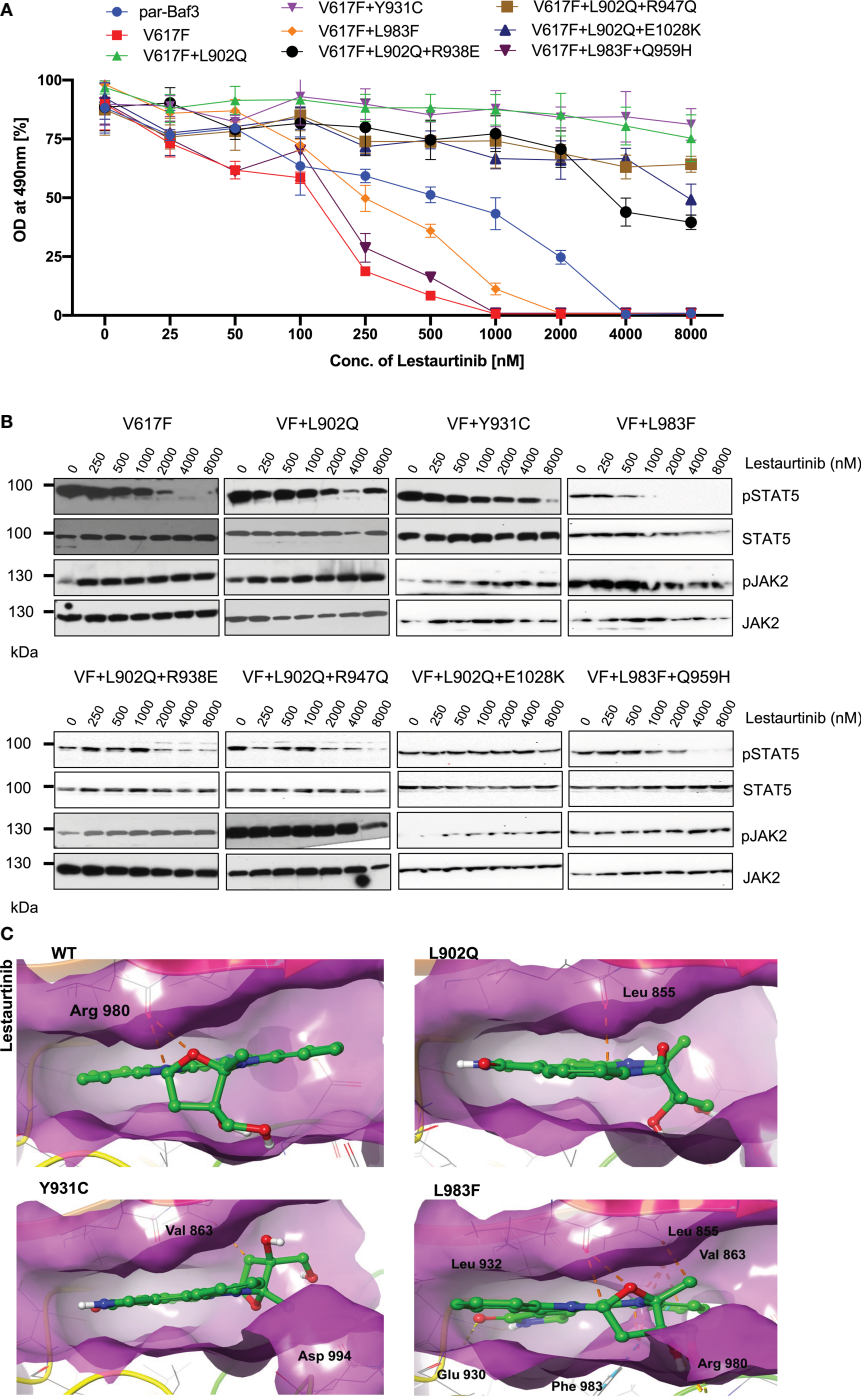
L983F and Y931C display sensitivity, whereas L902Q is resistant towards lestaurtinib: Mutations that emerged in the ruxolitinib screen were reengineered and expressed in the Ba/F3 cells. Cells were incubated in the presence of lestaurtinib. Proliferation was measured using 3-(4,5-dimethylthiazol-2-yl)-5-(3-carboxymethoxyphenyl)-2-(4-sulfophenyl)-2h-tetrazolium (MTS)- based method after incubation for 48hrs with the indicated concentrations of lestaurtinib **(A)**. Data is shown as mean ± standard deviation (SD) (n=3). OD – optical density. Immunoblot analysis of Ba/F3 cells expressing JAK2 mutants cultured with increasing concentrations of lestaurtinib (0, 250, 500, 1000, 2000, 4000 and 8000nM) for 4hrs and lysates were subjected to indicated antibodies **(B)**. A representative image of n=2 two independent experiments is shown. Molecular docking studies were performed using the Glide package of Schrodinger maestro software. The binding interactions of lestaurtinib (stick representation in green carbon) with the JAK2 Kinase domain and its mutants (secondary structure representation as a cartoon) are displayed. Hydrogen bonds are represented by dotted yellow lines, carbon centered hydrogen bonds are represented by dotted orange lines, pi-pi interactions are represented by dotted cyan lines and the amino acids responsible for these interactions are labelled in black. The binding interactions of lestaurtinib with wild type JAK2, L902Q, Y931C and L983F **(C)**.

Lestaurtinib is a potent inhibitor of FLT3, JAK2, JAK3, TRKA/B/B and VEGFR. However, previously it has been demonstrated that lestaurtinib inhibit JAK2-V617F expressing cells by inhibition of STAT5 (25). Based on these results, we hypothesized that lestaurtinib might inhibit ruxolitinib-resistant variants. Consistent with the previous reports, JAK2-V617F (IC50~89.9nM) was sensitive towards lestaurtinib indicating that this inhibitor is selective towards JAK2-V617F, however, analysis of other JAK2 variants results suggest that V617F+L902Q, V617F+Y931C, V617F+L902Q+R938E, V617F+L902Q+R947Q, and V617F+L902Q+E1028K are resistant to lestaurtinib (IC50 >4000nM). Lestaurtinib did not reduce the cell proliferation of these JAK2 variants at 8μM concentration (**Figure 5A**). Interestingly, lestaurtinib is potent against the V617F+L983F and V617F+L983F+Q959H (IC50~245nM and 117nM, respectively) (**Supplementary Table S1**). Consistent with the MTS-based cell proliferation data, biochemical data also showed inhibition of STAT5 in JAK2-V617F (**Figure 5B**) and increased JAK2 activation loop Tyr1007/1008 phosphorylation in the presence of lestaurtinib (**Figure 5B**). Whereas, V617F+L902Q, V617F+L902Q+R938E, V617F+L902Q+R947Q, and V617F+L902Q+E1028K did not show any decrease of STAT5 activation (**Figure 5B**). V617F+L983F, V617F+L983F+Q959H, and V617F+Y931C showed a decrease of STAT5 activation based on a dose-dependent manner, suggesting that these variants are inhibited at higher concentrations of lestaurtinib. In addition to compound mutations which are in combination with L902Q or L983F, we also established the single constitutes of R938E, R947Q, Q959H, and E1028K and found that these variants did not display resistance towards ruxolitinib, fedratinib, and lestaurtinib except R938E (**Supplementary Figures S2A-C**). R938E has a 2.5-fold increase of IC50 value towards ruxolitinib and lestaurtinib but no change in IC50 value against fedratinib. These results suggest that L902Q and L983F mutations are indispensable for strong drug resistance phenotypes in these variants.

Molecular docking analysis suggests that lestaurtinib forms two carbon-centered hydrogen bonds with wild-type JAK2 Arg 980 (**Figure 5C**; WT). The L902Q (with Leu 855), and Y931C (with Val 863) mutants showed single carbon-centered hydrogen bond interaction with lestaurtinib, indicating less binding affinity, which can be correlated with high IC50 values (**Figure 5C**; L902Q and Y931C). In contrast, for the L983F variant, lestaurtinib showed multiple hydrogen bond interactions (with Leu 932, Leu 855, Val 863, and Glu 930) and - interaction (with Phe 983), indicating stronger binding affinity and thus, low IC50 (**Supplementary Table S2**, **Figure 5C**; L983F). Taken together, computational data with the biochemical IC50 value data suggest that each inhibitor has a distinct affinity towards the kinase conformation, and alteration of these conformational states governs the drug binding state, which subsequently leads to the drug resistance phenotype. However, a complete three-dimensional structure of JAK2 is needed to explain the resistance phenotype of each variant involved in the modulation of the JAK2 kinase confirmation.

### 3.7 Type II JAK2 inhibitor CHZ 868 is potent towards the ruxolitinib-resistant variants

**Figure 6:**
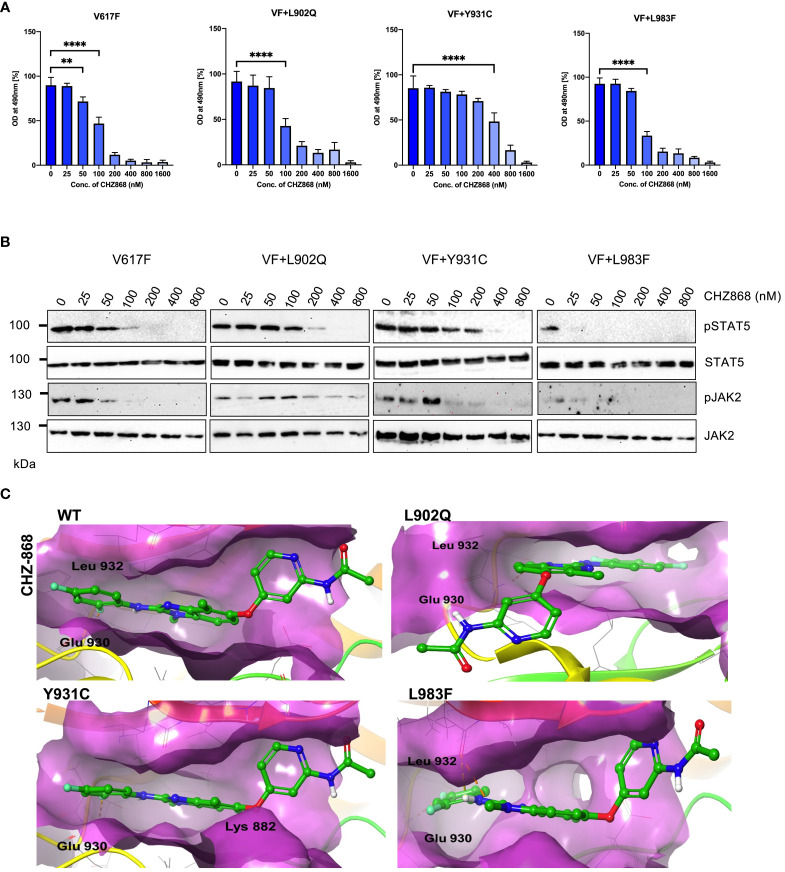
Type II JAK2 inhibitor CHZ-868 is more potent towards ruxolitinib resistant variants: Mutations that emerged in the ruxolitinib screen were reengineered and expressed in the Ba/F3 cells. Cells were incubated in the presence of type II JAK2 inhibitor CHZ-868 with the indicated concentrations **(A)**. Proliferation was measured using 3-(4,5-dimethylthiazol-2-yl)-5-(3-carboxymethoxyphenyl)-2-(4-sulfophenyl)-2h-tetrazolium (MTS)- based method after incubation for 48hrs with the indicated concentrations of CHZ-868. Data is shown as mean ± standard deviation (SD) (n=3). OD – optical density. **p<0.005, ****p<0.0001. Immunoblot analysis of Ba/F3 cells expressing JAK2 mutants cultured with increasing concentration of CHZ-868 (0, 25, 50, 100, 200, 400 and 800nM) for 4hrs and lysates were subjected to indicated antibodies **(B)**. A representative image of n=2 two independent experiments is shown. Molecular docking studies were performed using the Glide package of Schrodinger maestro software. The binding interactions of CHZ-868 (stick representation in green carbon) with the JAK2 Kinase domain and its mutants (secondary structure representation as a cartoon) are displayed. Hydrogen bonds are represented by dotted yellow lines, carbon centered hydrogen bonds are represented by dotted orange lines, pi-pi interactions are represented by dotted cyan lines and the amino acids responsible for these interactions are labelled in black. The binding interactions of CHZ-868 with wild type JAK2, L902Q, Y931C and L983F **(C)**.

Since type I inhibitors showed less efficiency toward the ruxolitinib-resistant variants, we decided to check the efficacy of type II JAK2 inhibitor CHZ-868 towards the ruxolitinib-resistant variants. Previously, it has been demonstrated that type II JAK2 inhibitor CHZ-868 showed remarkable efficacy in MPN mouse models compared to ruxolitinib (30). Based on our previous results, L902Q, Y931C, and L983F are the crucial resistant variants as other variants such as R938E, R947Q, Q959H and E1028K without L902Q or L983F did not alter their cellular IC50 value, thus, we focused on only L902Q, Y931C, and L983F variant’s sensitivity towards the CHZ-868. V617F+L902Q, which is resistant to ruxolitinib, fedratinib, and lestaurtinib, is sensitive towards CHZ-868 (**Figure 6A**; **Supplementary Table S1**). V617F+L983F is highly resistant towards ruxolitinib and is also sensitive towards the CHZ-868 (**Figure 6A**), suggesting that fedratinib and CHZ-868 should be beneficial to ruxolitinib refractory MPN patients. Surprisingly, V617F+Y931C, which is not a strong resistant variant towards the ruxolitinib, lestaurtinib, and fedratinib, is less sensitive towards CHZ-868 (**Figure 6A**), suggesting that type II inhibitors are more potent towards the resistant variants of type I inhibitors and also the mode of inhibitor interaction to the kinase domain determines the resistant phenotype. Analysis of JAK2 and STAT5 activation after CHZ-868 treatment results clearly showed a decrease of STAT5 and JAK2 activation in V617F, V617F+L902Q, and V617F+L983F (**Figure 6B**). V617F+Y931C displayed less sensitivity towards CHZ-868 by displaying the inhibition of STAT5 at 200nM concentration (**Figure 6B**).

Unlike type I JAK2 inhibitors that bind the ATP-binding site of the active JAK2 conformation, the type II JAK2 inhibitor CHZ-868 binds JAK2 allosterically in addition to the ATP-binding site of inactive JAK2 (41). Molecular docking analysis with CHZ-868 revealed that numerous hydrophobic interactions hold the drug with the residues Leu 855, Val 863, Ala 880, Val 911, Met 929, Leu 932, and Leu 983 in the binding pocket (**Figure 6C**; WT). In addition, CHZ-868 forms hydrogen bonds with Asp 994 and Glu 898, and van der Waal interacts with side chains of Leu 983, and Gly 993. CHZ-868 forms two hydrogen bond interactions in wild-type JAK2, L902Q, Y931C, and L983F variants (with Glu 930 and Leu 932) (**Figure 6C**). The mutations did not alter the binding interactions of CHZ-868 signifying similar binding affinities in all the variants (**Supplementary Table S2**).

### 3.8 JAK1-L1010F mutation (L983F homologous JAK2) drives strong ruxolitinib resistance phenotype

**Figure 7:**
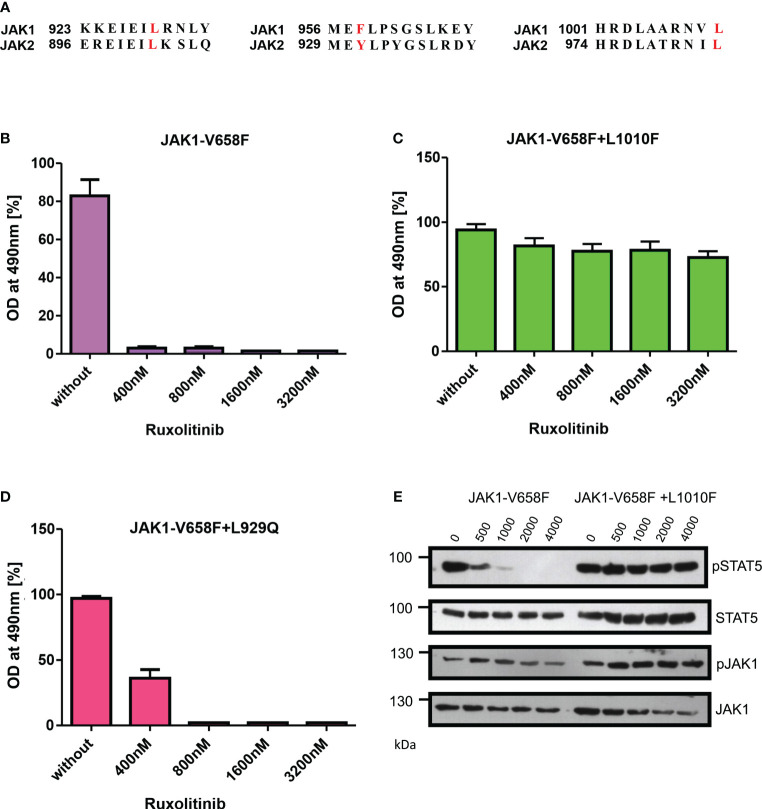
JAK2-L983F homologous mutation in JAK1-L1010F generates resistance phenotype towards ruxolitinib: Alignment of homologous regions in JAK2 and JAK1 **(A)**. JAK2 homologous mutations in JAK1 were recreated in JAK1-V658F using site-directed mutagenesis. Constructs were stably expressed in Ba/F3 cells. Proliferation was measured by MTS (3-(4,5-dimethylthiazol-2-yl)-5-(3-carboxymethoxyphenyl)-2-(4-sulfophenyl)-2h-tetrazolium-based method after incubation for 48hrs without and in the presence of indicated concentrations of ruxolitinib n=3 **(B–D)**. Immunoblot analysis of Ba/F3 cells expressing JAK1 mutants cultured with indicated concentrations of ruxolitinib for 4hrs and lysates were subjected to indicated antibodies **(E)**. A representative image of n=2 two independent experiments is shown.

To evaluate the role of ruxolitinib resistance in JAK1, we created the JAK2 homologous mutations in JAK1 and measured the ruxolitinib resistance (**Figure 7A**). In contrast to JAK2-V658F+L902Q which is strongly resistant towards ruxolitinib, the homologous mutation JAK1-V658F+L929Q gives only moderate resistance towards ruxolitinib suggesting structural variation among JAK-family kinases (**Figures 7B, D**). JAK2-V617F+L983F homologous mutation JAK1-V658F+L1010F gives strong ruxolitinib resistance (**Figures 7C, E**) suggesting that this mutation might pose a challenge in JAK1-mediated diseases as a resistant variant when patients are treated with JAK1 inhibitors.

### 3.9 HSP 90 inhibitors are effective therapeutic agents against the ruxolitinib-resistant variants

**Figure 8:**
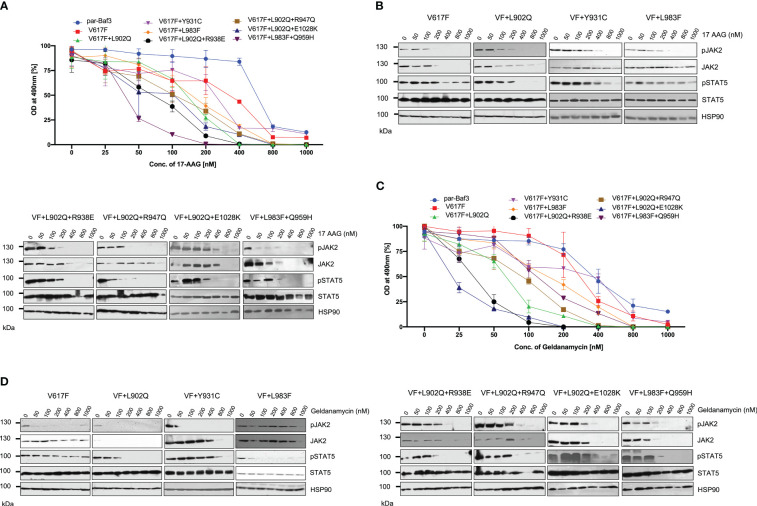
HSP 90 inhibitors target JAK2-V617F and overcome resistance to ATP-competitive inhibitors: Mutations that are identified in the ruxolitinib were stably expressed in the Ba/F3 cells and proliferation was measured using 3-(4,5-dimethylthiazol-2-yl)-5-(3-carboxymethoxyphenyl)-2-(4-sulfophenyl)-2h-tetrazolium (MTS)- based method after incubation for 48hrs with the indicated concentrations of 17-AAG **(A)**. Data is shown as mean ± standard deviation (SD) (n=3). OD – optical density. Immunoblot analysis of Ba/F3 cells expressing JAK2 mutants cultured with increasing concentrations of 17-AAG (0, 50, 100, 200, 400, 800 and 1000nM) for 4hrs and lysates were subjected to indicated antibodies **(B)**. A representative image of n=2 two independent experiments is shown. Similarly, Ba/F3 cells expressing JAK2 mutations were treated with indicated concentrations of Geldanamycin and measured the cell proliferation using the MTS-based method **(C)**. Data is shown as mean ± standard deviation (SD) (n=3). OD, optical density. Immunoblot analysis of Ba/F3 cells expressing JAK2 mutants cultured with increasing concentrations of Geldanamycin (0, 50, 100, 200, 400, 800 and 1000nM) for 4hrs and lysates were subjected to indicated antibodies **(D)**. A representative image of n=2 two independent experiments is shown.

JAK2 is the known client protein of HSP90 and inhibition of HSP90 by small molecular inhibitors leads to the degradation of both JAK2 wild-type and JAK2-V617F (42). HSP90 inhibitors were shown to be effective in the survival of JAK2-mediated MPNs in mice models and also shown as an alternative to genetic resistance mediated by JAK2 enzymatic inhibitors (42). We then hypothesized ruxolitinib resistant variants might also depend on the HSP90 pathway. Consistent to our hypothesis, HSP90 inhibitor 17-AAG treatment results suggest that V617F+L902Q, V617F+Y931C, V617F+L983F, V617F+L902Q+R938E, V617F+L902Q+R947Q, V617F+L902Q+E1028K and V617F+L983F+Q959H are sensitive to 17-AAG (**Figure 8A**). These results indicate that these mutants are dependent on the HSP90 for their folding. To know that downregulation of JAK2 protein leads to the decrease of cell proliferation, we performed biochemical analysis on these mutant JAK2 cells and found that ruxolitinib-resistant variants are sensitive towards 17-AAG and treatment of the cells with 17-AAG leads to the downregulation of JAK2 protein and decrease of STAT5 activation (**Figure 8B**).

To confirm that HSP90 inhibition leads to the inhibition of cell proliferation of mutant JAK2 cells, we used another potent HSP90 inhibitor, geldanamycin. Similar to the 17-AAG results, geldanamycin is also effective in the inhibition of cell growth both in JAK2-V617F cells and drug resistant mutants (**Figure 8C**). Western blot data also suggested that treatment of the cells with geldanamycin leads to the downregulation of the JAK2 protein more effectively in the drug resistant mutants compared to JAK2-V617F and also decreased the STAT5 activation (**Figure 8D**).

## 4 Discussion

JAK2-V617F mutation is frequently reported in myeloproliferative neoplasms (MPNs) such as polycythemia vera, essential thrombocythemia, and primary myelofibrosis (3–5, 7). Constitutive JAK2 signaling is also involved in several solid tumors and other lymphoid malignancies (15, 16, 43). The occurrence of JAK2 variants in several malignancies suggests that therapeutic inhibition of JAK2 is important. Two JAK2 inhibitors, ruxolitinib and fedratinib, have been approved for patients with intermediate and high-risk myelofibrosis. It has been demonstrated that in chronic myelogenous leukemia (CML) and gastrointestinal stromal tumors (GIST), acquired resistance to imatinib is due to the emergence of secondary kinase domain mutations in target kinase. In the case of *BCR::ABL*, more than 50 different mutations have been described that confer drug resistance in CML patients (44). Results from imatinib resistance in CML suggested sequential treatment with TKIs and approval of second-generation ABL kinase inhibitors. However, in the case of JAK2, no drug-resistant variants are reported in MPN patients even though they are resistant (persistent) to JAK2 inhibitors. The mechanism responsible for JAK2-inhibitor persistence in MPN patients is not understood well. One possible explanation for the absence of the point mutations in the kinase domain of the JAK2 against the ruxolitinib is due to the compromisation of the kinase function. Thus, it is essential to identify the critical residues that mediate the inhibitor resistance without compromisation of the kinase function. In this study, we have identified seven different mutations in the kinase domain of JAK2 that conferred strong resistance towards ruxolitinib. We functionally evaluated these residues in the kinase domain function and resistance towards the type I and type II JAK2 inhibitors (**Figures 3**–**6**). All the mutations identified in our cell-based screen showed similar transformation ability and JAK2 and STAT5 activation except Y931C. JAK2-Y931C gives enhanced cell growth compared to other JAK2 variants. This result is in line with Downes et al. study using the *ATF7IP::JAK2* model where ruxolitinib resistance was mediated by Y931C exchange, which also induced the enhanced cell proliferation and JAK/STAT signaling pathway (45). In addition to this finding, the occurrence of only seven resistant mutations in our screen is in line with other studies by Deshpande et al. and Kesarvani et al. study, which demonstrated the limited repertoire of JAK2 mutations against the JAK2-inhibitor resistance in contrast to ABL inhibitors (40, 46).

In the case of c-Kit mediated drug resistance, the acquisition of point mutation (D816V) leads to a change in the equilibrium of the kinase towards the active conformation that results in the activation of the c-Kit (47). Similarly, FLT3-D835 mutation is known to cause activation of the FLT3 and resistance towards the FLT3 inhibitors (48). In the case of JAK2, the drug-resistant mutations L902Q and L983F did not activate the JAK2 and STAT5 without V617F mutation, suggesting that V617F mutation is indispensable for the activation of JAK2-STAT5 and cytokine-independent growth. Similar to our results, Hornakoa et al. study identified several mutations in JAK1 by cytokine deprivation. In this study, they found F958C mutation in cytokine-independent clones. Interestingly, JAK1-F958C is resistant to JAK inhibitors CMP6 and ruxolitinib (49). JAK1-F958 is homologous to JAK2-Y931, which we observed as a drug-resistant variant in our screen. In addition, two other publications used random mutagenesis screens and observed JAK2-Y931C mutation against ruxolitinib and BVB808 (37, 50). Interestingly, a more comprehensive study by Keserawani et al. detected 211 amino acid substitutions in whole JAK2 protein, which give cross-resistance against the panel of JAK inhibitors by using randomly mutagenized JAK2-V617F expressed in Ba/F3 cells (40). In line with their study, our chemical mutagenesis screen also detected JAK2 mutations at residues L902, Y931, R938, L983, and E1028, which give strong resistance against ruxolitinib. In our study, we found only seven different mutations in our cell-based screen. This is in contrast to *BCR::ABL*, where 112 distinct amino acid substitutions affect 90 residues in *BCR::ABL* (29). We used a high concentration of ruxolitinib, such as 4µM (IC50~20 times) and 8µM (IC50~40 times) in our cell-based screen, which might lead to the escape of moderate drug-resistant clones. However, the ruxolitinib screen performed with low concentration did not yield any kinase domain mutations (unpublished data). One possibility for this is a heterodimeric association of JAK1 and JAK2 or TYK2, as reported by Koppiker et al. study (51). We found a 45-kDa novel JAK2 variant that alters kinase domain structure and generates ruxolitinib resistance in Ba/F3 cell line model system without ENU treatment suggesting that novel short form of JAK2 variants need to be analyzed in ruxolitinib resistant MPN patients (52).

In this study, we identified JAK2-V617F+L902Q, JAK2-V617F+Y931C, and JAK2-V617F+L983F are the most frequent ruxolitinib-resistant mutation. These mutations conferred resistance to ruxolitinib and cross-resistance to lestaurtinib and fedratinib. JAK2-V617F+L983F and V617F+L983F+Q959H identified in the ruxolitinib screen conferred cross-resistance to other JAK2 inhibitors but were very sensitive towards fedratinib and CHZ-868. In line with this study, Kesarvani et al. demonstrated that fedratinib binds to both ATP and substrate binding sites. Due to the ability of ATP-binding and substrate binding, fedratinib showed a complete lack of genetic resistance (40). Our results suggest that ruxolitinib-resistant variant L983F poses a significant challenge in MPN patients in the future. However, fedratinib could potentially be used in these patients. In contrast to type I JAK2 inhibitors, which bind JAK2 in a phosphorylated state, type II JAK2 inhibitor CHZ-868 which binds JAK2 in unphosphorylated form and is active against the JAK2-V617F+L902Q, JAK2-V617F+Y931 and JAK2-V617F+L983. In addition, CHZ-868 did not increase the JAK2 activation loop Tyr 1007/1008 phosphorylation compared to type I inhibitors. These results are in line with Downes et al. study, where in *ATF7IP*::*JAK2* mediated acute lymphoblastic leukemia (ALL), CHZ-868 is potent against the ruxolitinib resistant variants such as Y931C and L983F (45). These observations suggest that the development of type II JAK2 inhibitors whose mode of interaction is different from ruxolitinib is essential to guide future rational drug-designing strategies to overcome drug resistance in JAK2-mediated diseases. In this study, we also created the ruxolitinib-resistant homologous mutations in JAK1 and identified that L1010F (L983F in JAK2) mutation in JAK1 mediates the ruxolitinib resistance. Previously, it was well established in AML that resistance against the FLT3-TKIs is also due to the acquisition of activation mutations in JAK1 and JAK3 (53). Based on these observations, it is important to perform combination treatment approaches such as FLT3 inhibitors (midostaurin or gliteritinib) in combination with JAK-family kinase inhibitors (ruxolitinib or momelotinib) to achieve better therapeutic response in AML. It is also noteworthy that the L1010F mutation in JAK1 might pose a challenge as this variant drives the resistance against JAK1 inhibitors. However, in our study, we found that twenty percent of clones did not yield any mutation in the JAK2 kinase domain but were still resistant to ruxolitinib. These clones might have acquired mutations in other signaling pathway genes, such as JAK1/TYK2. Therefore, it is noteworthy to sequence the resistant clones for other JAK-family kinases in order to rule out the possibility that ruxolitinib resistance in these clones is not due to the acquisition of mutations in the kinase domain of other JAK-family members.

Similar to our study, a study by Deshpande et al. using the random mutagenesis method also identified R938L as a ruxolitinib-resistant mutation (46). They hypothesize that the R938L mutation changes the main chain conformation that affects the receptor binding and affinity due to their proximity to the binding pocket. In our screen, we saw R938E instead of R938L. Substitution of Asp at this position is not enough to give strong drug resistance compared to Leu with a more hydrophobic nature. Although we used JAK2-V617F Ba/F3 cells to predict the ruxolitinib resistant mutations, one must focus on other JAK2 variants such as point mutations in JAK2 other than V617F (exon 12 mutations) (9), additional JAK2 point mutations in pediatric or adult B-ALL (54, 55), or active form of JAK2 including *TEL::JAK2* (14), *PCM::JAK2* (56) and *BCR::JAK2* (57) to predict resistance mechanisms against the JAK inhibitors.

In cell culture-based experiments we show that the resistance to ruxolitinib is mediated by secondary mutations in the kinase domain of JAK2, however, these secondary mutations are yet to be identified in MPN patients. While JAK2 inhibitor resistance at molecular level is ill-defined, there is increasing evidence that ruxolitinib treatment persistence in MPN patients may be due to non-JAK2 mutations, such as activating mutations of RAS, MEK and AKT (58). Using a whole exome sequencing and single cell genotyping approach, Mylonas et al. showed that mutations in RAS/RTK pathway genes were enriched in one-third of myelofibrosis patients during ruxolitinib therapy (59). Utilizing an MPN mouse model, Stivala et al. described that cell extrinsic mechanisms could provide survival signaling, such as activation of PDGFR signaling via its ligand PDGF-BB by ruxolitinib treatment (60). In addition, the bone marrow microenvironment might also be involved in ruxolitinib sensitivity, as myelofibrosis patients display an inflammatory phenotype. Fisher et al. characterized the expression of certain cytokines, whose production is not inhibited by ruxolitinib such as TNF, IL-6, IL-8 and IL-10 using mass cytometry analysis. Accordingly, monocytes, which are the major cellular source of many cytokines, affect disease-propagating stem and progenitor cells suggesting the involvement of the bone marrow microenvironment in ruxolitinib sensitivity (61).

Computational modeling studies further provide evidence for the ruxolitinib resistance in L902Q, Y931C, and L983F. In many studies, an isolated JH1 domain is used to perform inhibitor-kinase interaction to define the residues that mediate the drug resistance phenotype. However, it is also essential to consider the performance of computational modeling together with the JH2 domain, as V617F is located in this domain, affecting the conformation of the kinase domain. This could be one reason for certain kinase domain residues whose binding energies are not significantly altered in molecular docking studies, however, biochemical results showed a strong resistance phenotype.

Heat shock protein 90 (HSP90) is a molecular chaperone that plays a major role in the maturation of several client proteins, such as fusion kinases and oncogenic proteins. Inhibition of the HSP90 pathway has been proven as a therapeutic strategy for the treatment of myeloma and other cancers (62). Recently, it has been demonstrated that JAK2 is the client protein of HSP90. Treatment of JAK2-V617F cells with HSP90 inhibitors leads to the downregulation of the JAK2 protein and HSP90 inhibitors are active in mice models of JAK2-V617F and MPL-mediated MPNs (42). In this study, we also demonstrated that HSP90 inhibition overcomes the ruxolitinib-resistant mutations. Both 17-AAG and geldanamycin showed inhibition of ruxolitinib-resistant mutations more effectively than JAK2-V617F cells. We observed that JAK2-V617F+L902Q, JAK2-V617F+Y931C, and JAK2-V617F+L983F mutations are sensitive to HSP90 inhibitors compared with cells lacking resistant mutation (**Figures 7A, B**). This observation suggested that drug-resistant mutations depend more on the HSP90 activity than non-resistant mutations (JAK2-V617F). Taken together, our study also provides a rationale that HSP90 inhibitors are the possible therapeutic agents in the case of JAK2 kinase inhibitor-resistant mutations and suggests the importance of the clinical evolution of HSP90 inhibitors in drug-resistant MPN patients.

One major limitation of our study is the drug-resistance screen was performed using murine Ba/F3 cell lines instead of human MPN cell lines. However, the ruxolitinib resistance screen performed with HEL (JAK2-V617F^+^) cell lines did not yield any drug-resistant clones suggesting that the occurrence of drug resistance mechanisms in the human cell lines might be complex. Previously, using Ba/F3 cells expressing oncogenes such as *BCR::ABL*, FLT3-ITD, and *FIP1L1::PDGFRA* we have established drug-resistant variants against the TKIs. This cell-based screening method faithfully reproduces the mutations reported in the clinical setting and helps in treatment decisions for CML, AML and CEL patients (31, 36, 63–65). Together with these results, we believe that the Ba/F3 cell line model is informative as it represents the clinical situation in leukemia patients. Nevertheless, our study will be helpful in using drug-resistant JAK2/JAK1 variants as a tool for the development of novel JAK-family kinase inhibitors with high potency.

## Data availability statement

The raw data supporting the conclusions of this article will be made available by the authors, without undue reservation.

## Ethics statement

Ethical approval was not required for this study as we used only commercially available cell lines.

## Author contributions

SG: Conceptualization, Data curation, Formal analysis, Funding acquisition, Investigation, Methodology, Project administration, Resources, Software, Supervision, Validation, Visualization, Writing – original draft, Writing – review & editing. GP: Data curation, Formal analysis, Investigation, Methodology, Validation, Visualization, Writing – review & editing. JO: Data curation, Methodology, Validation, Writing – review & editing. DD: Data curation, Formal analysis, Methodology, Software, Writing – review & editing. VB: Data curation, Formal analysis, Writing – review & editing. JD: Funding acquisition, Resources, Writing – review & editing. NvB: Funding acquisition, Resources, Writing – review & editing.
