# Supplementary material for: Type II mode of JAK2 inhibition and destabilization are potential therapeutic approaches against the ruxolitinib resistance driven myeloproliferative neoplasms
Source: Front Oncol. 2024 Jul 18;14:1430833. doi: 10.3389/fonc.2024.1430833 (PMC11291247; doi:10.3389/fonc.2024.1430833)
Supplement: Supplementary file 1 [file DataSheet_1.docx]

Supplementary Material

**Supplementary Table S1**: IC50 profile of JAK2 mutations against the ruxolitinib, fedratinib, lestaurtinib and CHZ-868.

| Mutation | IC50 value  Ruxolitinib (nM) | IC50 value  Fedratinib  (nM) | IC50 value  Lestauritinib (nM) | IC50 value CHZ-868  (nM) |
| --- | --- | --- | --- | --- |
| V617F | 182.8 + 15 | 172.3 + 38 | 89.95 + 53 | 50 + 25 |
| V617F+L902Q | >6000 | >1000 | >4000 | 46 + 35 |
| V617F+Y931C | >4000 | 145 + 35 | >4000 | 200 + 34 |
| V617F+L983F | >8000 | 88.55 + 28 | 245.5 + 45 | 45 + 24 |
| V617F+L902Q+R938E | >6000 | >1000 | >4000 | n.d. |
| V617F+L902Q+R947Q | >6000 | >1000 | >4000 | n.d. |
| V617F+L983F+Q959H | >8000 | 91.3 + 45 | 117 + 42 | n.d. |
| V617F+L902Q+E1028K | >6000 | >1000 | >4000 | n.d. |

(n.d.-not determined)

**Supplementary Table S2**: Scoring profiles of selected drugs with the wild type and mutant JAK2.

|  | Ruxolitinib | Fedratinib | Lestaurtinib | CHZ-868 |
| --- | --- | --- | --- | --- |
| **Wild-type** |  |  |  |  |
| Glide Score | -9.130 | -9.710 | -6.909 | -5.757 |
| Binding Energy | -46.048 | -56.863 | -41.640 | -46.408 |
| **L902Q** |  |  |  |  |
| Glide Score | -8.997 | -7.518 | -5.974 | -5.137 |
| Binding Energy | -44.039 | -53.946 | -23.859 | -50.940 |
| **Y931C** |  |  |  |  |
| Glide Score | -8.715 | -7.085 | -5.690 | -5.319 |
| Binding Energy | -43.494 | -54.603 | -43.440 | -46.754 |
| **L983F** |  |  |  |  |
| Glide Score | -9.264 | -7.834 | -10.460 | -6.132 |
| Binding Energy | -42.784 | -51.871 | -28.229 | -49.960 |

**Supplementary Figure 1**

**Supplementary Figure 1:** **JAK2-V617F exchange is indispensable for oncogenic activation of JAK2**: JAK2-L902Q, JAK2-L983F were cloned both in wild-type JAK2 and JAK2-V617F background and measured the transformation ability in the absence of IL-3. JAK2-V617F+L902Q and JAK2-V617F+L983F only transform the Ba/F3 cells, whereas JAK2-L902Q and JAK2-L983F fail to transform the Ba/F3 cells (A and B).

**Supplementary Figure 2**

**Supplementary Figure 2**: **Single JAK2 variants without L902Q and L983F exchange failed to induce resistance phenotype towards JAK2 inhibitors:** JAK2 mutants that were identified with the ruxolitinib screen only single constituents of compound mutations were recreated in JAK2-V617F using site-directed mutagenesis. Constructs were stably expressed in Ba/F3 cells. Proliferation was measured using (3-(4,5-dimethylthiazol-2-yl)-5-(3-carboxymethoxyphenyl)-2-(4-sulfophenyl)-2h-tetrazolium (MTS)- based method after incubation for 48hrs without and in the presence of increasing concentration of the inhibitors ruxolitinib(A), fedratinib (B), lestaurtinib (C). OD indicates optical density. Values are expressed as the mean of triplicates.
